# Alpha-Melanocyte Stimulating Hormone Protects against Cytokine-Induced Barrier Damage in Caco-2 Intestinal Epithelial Monolayers

**DOI:** 10.1371/journal.pone.0170537

**Published:** 2017-01-19

**Authors:** Judit Váradi, András Harazin, Ferenc Fenyvesi, Katalin Réti-Nagy, Péter Gogolák, György Vámosi, Ildikó Bácskay, Pálma Fehér, Zoltán Ujhelyi, Gábor Vasvári, Eszter Róka, David Haines, Mária A. Deli, Miklós Vecsernyés

**Affiliations:** 1 Department of Pharmaceutical Technology, Faculty of Pharmacy, University of Debrecen, Debrecen, Hungary; 2 Institute of Biophysics, Biological Research Centre, Hungarian Academy of Sciences, Szeged, Hungary; 3 Department of Immunology, Faculty of Medicine, University of Debrecen, Debrecen, Hungary; 4 Department of Biophysics and Cell Biology, Faculty of Medicine, University of Debrecen, Debrecen, Hungary; 5 Department of Pharmacology, Faculty of Pharmacy, University of Debrecen, Debrecen, Hungary; University of Szeged, HUNGARY

## Abstract

Alpha-melanocyte-stimulating hormone (α-MSH) is a potent anti-inflammatory peptide with cytoprotective effect in various tissues. The present investigation demonstrates the ability of α-MSH to interact with intestinal epithelial cell monolayers and mitigate inflammatory processes of the epithelial barrier. The protective effect of α-MSH was studied on Caco-2 human intestinal epithelial monolayers, which were disrupted by exposure to tumor necrosis factor-α and interleukin-1β. The barrier integrity was assessed by measuring transepithelial electric resistance (TEER) and permeability for marker molecules. Caco-2 monolayers were evaluated by immunohistochemistry for expression of melanocortin-1 receptor and tight junction proteins ZO-1 and claudin-4. The activation of nuclear factor kappa beta (NF-κB) was detected by fluorescence microscopy and inflammatory cytokine expression was assessed by flow cytometric bead array cytokine assay. Exposure of Caco-2 monolayers to proinflammatory cytokines lowered TEER and increased permeability for fluorescein and albumin, which was accompanied by changes in ZO-1 and claudin-4 immunostaining. α-MSH was able to prevent inflammation-associated decrease of TEER in a dose-dependent manner and reduce the increased permeability for paracellular marker fluorescein. Further immunohistochemistry analysis revealed proinflammatory cytokine induced translocation of the NF-κB p65 subunit into Caco-2 cell nuclei, which was inhibited by α-MSH. As a result the IL-6 and IL-8 production of Caco-2 monolayers were also decreased with different patterns by the addition of α-MSH to the culture medium. In conclusion, Caco-2 cells showed a positive immunostaining for melanocortin-1 receptor and α-MSH protected Caco-2 cells against inflammatory barrier dysfunction and inflammatory activation induced by tumor necrosis factor-α and interleukin-1β cytokines.

## Introduction

Epithelial cells are key components of the intestinal barrier by forming tight junctions (TJ) sealing the paracellular cleft, thus restricting free flux of cells and molecules from the gut to the blood. Dysfunction of the epithelial barrier is a common feature in inflammatory diseases of the gastrointestinal system [1]. The damage of the protective epithelial barrier contributes to the pathomechanism and both local and systemic inflammation. Proinflammatory cytokines tumor necrosis factor-α (TNF- α) and interleukin-1β (IL-1 β) are overexpressed in inflammatory bowel diseases and directly damage the intestinal barrier including the interepithelial TJs [1]. Cell culture models of intestinal epithelium are widely used in the characterization of gut disease pathomechanisms, and to evaluate selected pharmacotherapies. The Caco-2 human intestinal epithelial cell line is a well-characterized model to study intestinal absorption processes [2], and is also used to investigate intestinal inflammation [3–6].

Since TNF-α and IL-1β are pathogenic factors in intestinal inflammation, they are used in both animal and culture models to induce epithelial cell inflammation and barrier opening. These cytokines induce initiation and amplification of inflammatory cellular processes which alter Caco-2 function, such as cell layer permeability, in ways that can be used as the model of inflamed bowel epithelium [7–9]. Treatment of Caco-2 cells with TNF-α or IL-1β decrease the electrical resistance of monolayers and increase IL-8 production indicating epithelial barrier opening and inflammatory response [8,10,11]. In our previous study we described, that claudin-4, a sealing claudin, is the most expressed member of the claudin family after claudin-1 in Caco-2 cells [12]. Claudin-4 was described as an important element of the intestinal barrier in both colon tissue of mice and Caco-2 cells with a significant downregulation in inflammation [13].

A prominent member of the melanocortin system, α-MSH, regulates crucial aspects of not only melanogenesis but also inflammation in various cell types [14]. The antiinflammatory effects of α-MSH are mediated by the inhibition of NF-κB induced inflammatory processes, like activation and proliferation of lymphocytes, and proinflammatory cytokine production [15,16]. Due to this protective action the therapeutical potential of α-MSH has been widely examined in immune-mediated pathologies, like allergic and inflammatory diseases of the skin and lung, ocular inflammation, arthritis, and inflammatory bowel disease [16]. The antiinflammatory effects of α-MSH have been examined in animal models of intestinal injury. In a rat model of chemically induced acute and chronic colitis α-MSH reduced pathological weight loss, fecal blood, TNF-α and nitric oxide production in colon tissue [17] and macroscopic colitis lesions [18]. Protective effect of α-MSH was also described in rat models of intestinal ischemia/reperfusion, where NF-κB induced inflammation has a central role in the pathomechanism [19,20].

The immunomodulatory action of α-MSH is regulated by melanocortin receptors MC1, MC3, MC4 and MC5 [16]. The presence of MC1R, the most important receptor responsible for mediating the antiinflammatory effects of α-MSH, was demonstrated on intestinal epithelium in mice [21]. The crucial role of this receptor in inflammatory gut disease was demonstrated in sophisticated mouse models, where the absence of a functional MC1R resulted in the aggravation of different types of experimental colitis indicating the protective role of the α-MSH-MCR1 pathway on non-hematopoietic cells [21].

Based on these data we hypothesized a direct protective action of α-MSH on cytokine-induced barrier dysfunction and inflammatory activation in the human Caco-2 epithelial cell line, which has not been investigated, yet. Therefore the aims of the present study were: (i) demonstration of the presence of MC1R on Caco-2 epithelial cells, (ii) testing the effects of α-MSH on TNF-α and IL-1β treatment induced barrier damage by measurement of transepithelial electric resistance (TEER) and permeability for marker molecules, and visualization of TJ proteins ZO-1 and claudin-4, (iii) investigating the inhibitory action of α-MSH on cytokine-induced inflammatory response, including NF-κB nuclear translocation, and polarized secretion of proinflammatory cytokines IL-6 and IL-8.

## Materials and Methods

### Materials

All reagents were purchased from Sigma-Aldrich Corporation (subsidiary of Merck KGaA, Darmstadt, Germany) unless otherwise indicated.

### Cell culture

Human Caco-2 intestinal epithelial cells (#86010202) purchased directly from European Collection of Authenticated Cell Cultures, UK, were cultured in Dulbecco’s Minimum Essential Medium (DMEM) supplemented with 10% fetal bovine serum [22]. Cell monolayers (10–40 passages) were grown on permeable polycarbonate inserts (Transwell^®^, Corning, Lowell, MA, USA; 0.4 μm pore size, 1.12 cm^2^ surface) in CO_2_ incubator at 37°C. Cell seeding density was 2 × 10^5^ cells/insert. Culture medium was replaced with fresh medium every two or three days. During the 21-day culture period Caco-2 cells formed fully differentiated, confluent monolayers with apical-basal polarization. Differentiated and polarized cell layers showing high TEER values were used for experiments, as described in our previous study [23].

### Treatments

To induce barrier damage differentiated Caco-2 cell cultures were treated with a combination of the proinflammatory cytokines TNF-α (50 ng/ml) and IL-1β (25 ng/ml) for 24 hours. The control group received culture medium only (negative control). To test the protective effect of α-MSH the following concentrations were used: 10^−4^ M, 10^−8^ M, 10^−12^ M, 10^−16^ M.

### Measurement of transepithelial electrical resistance

The formation of tight paracellular barrier in Caco-2 layers was monitored by the development of high transepithelial electrical resistance (TEER) and measured with Millicell-ERS volt-ohm meter (Millipore, USA) using chopstick electrodes. Resistance was expressed to the surface of the Transwell filters (Ω × cm^2^) and the TEER of cell-free inserts was subtracted from these values.

### Measurement of permeability for marker molecules

Cell culture inserts (surface: 0.33 cm^2^) containing confluent and differentiated Caco-2 cells were transferred to plates containing 530 μl Ringer-Hepes buffer (150 mM NaCl, 2.2 mM CaCl_2_, 0.5 mM MgCl_2_, 5.2 mM KCl, 6 mM NaHCO_3_, 2.8 mM glucose and 5 mM Hepes, pH 7.4) in the basal (lower) compartments. In the apical (upper) compartments culture medium was replaced by 70 μl buffer containing albumin (10 mg/ml; Mw: 67 kDa) in complex with Evans blue (167.5 μg/ml) and sodium fluorescein (10 μg/ml, Mw: 376 Da). The plates were kept at 37°C in an incubator with 5% CO_2_ for 1 hour on a rocking platform (100 rpm). After the incubation samples from the upper and lower compartments were collected and the concentrations of the marker molecules were determined by a fluorescence multiwell plate reader (Fluostar Optima, BMG Labtechnologies, Germany; excitation wavelength: 485 nm, emission wavelength: 535 nm for fluorescein and excitation wavelength: 584 nm, emission wavelength: 680 nm for Evans-blue labeled albumin). The apparent permeability coefficients (P_app_) were calculated by the following equation [24].

$${\text{P}}_{\text{app}}\;=\frac{{\left[C\right]}_{B}\;\times \;V_{B}}{A\times {\left[C\right]}_{A}\times t}$$

where [C]_B_ is the concentration of the tracer in the basal (acceptor) compartment after 1 hour, [C]_A_ is the concentration of the tracer in the apical (donor) compartment at 0 hour, V_B_ is the volume of the basal compartment (530 μl) and A is the surface area available for permeability (0.33 cm^2^).

### Immunohistochemistry

#### Melanocortin-1 receptor

Caco-2 cell monolayers grown on polycarbonate filters were washed in phosphate buffered saline (PBS) and fixed with 4% paraformaldehyde solution for 20 minutes. Cell membranes were permeabilized with 0.2% Triton-X for 10 minutes. Non-specific binding sites were blocked with FBS for 30 minutes. A rabbit anti-human MC1R antibody (M9193 Sigma-Aldrich, AB_212630, 1 mg/ml) was used for primary labelling of the target antigen, followed by secondary labelling with Alexa Fluor 488 conjugated to goat-anti-rabbit IgG (A-11008, Thermo Fisher Scientific, Waltham, MA, USA, AB_143165, 2 mg/ml). Cell nuclei were labelled with propidium iodide. The samples were thoroughly washed with PBS after each step. Stainings were visualized by an Olympus FV1000 confocal microscope using an UPLSAPO 60× (NA 1.35) oil immersion objective. Excitation of Alexa Fluor 488 (conjugated to anti-MC1R) was accomplished using the 488 nm line of an Ar ion laser; with an emission spectrum of 500–530 nm. Excitation of propidium iodide was done using a 543 nm HeNe laser, with an emission spectrum of 555–635 nm. Excitation of Alexa Fluor 647 (labeling anti-MHC I W6/32 mAb) was done using a 633 nm HeNe laser, with an emission spectrum of 655-755nm. Line-by-line alternating illumination with the different lasers was used to minimize crosstalk. Images (256×256-pixels; pixel size: 0.103 μm; pixel time: 4 μs) were collected and 4× averaging was applied to reduce background noise. Image stacks were recorded with a step size of 0.8 μm and an optical slice thickness of 0.88 μm (pinhole: 93 μm). Projection images of three adjacent optical sections were generated at the basal and at the apical side of the cells using the FV1000 software for the microscope. 3D image reconstructions of the cells were generated from image stacks. Transverse views of cells were used to visualize receptor expression levels of the apical and basal cell surfaces.

### ZO-1 and claudin-4 tight junction proteins and nuclear factor kappa B (NF-κB) activation

For these experiments, Caco-2 cells were seeded onto sterile uncoated microscope slides at a density of 5 × 10^4^ cells/slide, and cultured until the formation of monolayers. Caco-2 cultures were divided into three groups: the first group received culture medium only and served as negative controls. The second group was treated with IL-1β and TNF-α; and the third group received 10^−8^ M α-MSH in addition to cytokines. Treatments lasted 24 hours for the tight junction protein stainings, and for 30 minutes for p65 protein immunostaining, which is an indicator of NF-κB activation and nuclear localization. For ZO-1 and claudin-4 immunostainings cells were fixed and permeabilized with acetone/methanol 1:1. Primary staining was accomplished using rabbit anti-human ZO-1, or claudin-4 antibodies (40–2200 and PA5-16875, AB_2533456 and AB_10978522, 0.5 μg/ml), followed by secondary labelling with Alexa Fluor 488 conjugated goat-anti-rabbit IgG (all antibodies from Thermo Fisher Scientific, MA USA). For p65 staining cells were fixed with ice-cold methanol-acetone (50 v/v %) for 10 minutes. Nonspecific antibody binding sites were blocked with FBS for 15 minutes. For primary labelling of NF-κB p65 subunit rabbit anti-human p65 (sc-372, Santa Cruz Biotechnology, AB_632037, 100 μg/ml) was used followed by secondary staining with Alexa Fluor 488-conjugated goat-anti-rabbit IgG and Hoechst 33342. For both TJ proteins and NF-κB stainings samples were observed by Zeiss Axio Scope.A1 fluorescent microscope (HBO 100 lamp) (Carl Zeiss Microimaging GmbH, Göttingen, Germany). Images were analyzed with ZEN 2012 v.1.1.0.0. software (Carl Zeiss Microscopy GmbH, Göttingen, Germany), and for the NF-κB stainings the ratio of nuclear and perinuclear fluorescence intensity was calculated. Specificity of all immunostainings was checked by incubating the cells with secondary antibodies only, and no background stainings were found.

### Flow cytometric bead array cytokine assay

Production of the inflammatory cytokines IL-6 and IL-8 by Caco-2 cells were measured in cell culture medium using the multiplexed flow cytometric bead array (CBA) Human Inflammatory Cytokines Kit (BD Biosciences, San Jose, CA, USA). For this assay 50 μl culture medium samples were collected at 4 and 24 hours timepoints from the apical and basal compartments of culture inserts with confluent Caco-2 monolayers treated with culture medium, cytokines and /or α-MSH. The CBA kits were used according to the manufacturer’s instructions. The collected samples were diluted 2 times in the kit’s assay buffer. Diluted samples or appropriate cytokine standards (50 μl) were added to equal amounts of fluorescent cytokine capture bead suspension (50 μl), and human inflammatory cytokine-phycoerythrin detection reagent (50 μl) on multi-well filter microplates. The plates were incubated for 2 hours on a microplate shaker at room temperature, followed by washing with a vacuum filtration manifold. To each well 120 μl assay buffer was added and cytokine content of each sample was measured by FACS array cytometry (BD Biosciences). Outcome data were analyzed using Flow Cytomix Pro 2.3 software.

### Statistical analysis

For statistical analysis SigmaStat software (version 3.1; SPSS Inc.) and GraphPad Prism 5.0 software (GraphPad Software Inc., USA) were used. Data are presented as means±S.D. Comparison of groups was performed using ANOVA and Dunnett or Bonferroni tests.

Differences were considered significant at *P*<0.05.

## Results

### Expression of melanocortin-1 receptor on Caco-2 cells

**Fig 1:**
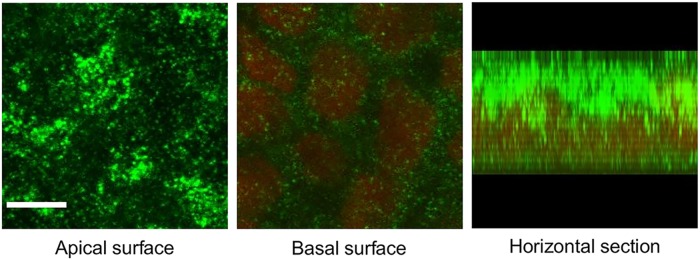
Melanocortin-1 receptor (MC1R) expression in Caco-2 cells. Cells were grown into confluent monolayers for 21 days on culture inserts and immunostained for MC1R. Nuclei were labeled with propidium iodide. Optical sections prepared by a confocal microscope show MC1R expression levels at the apical and basal surfaces, and a digitized horizontal section of the cells. Green: MC1R labeling; red: cell nuclei. Scale bar: 5 μm.

MC1R expression was detectable on both the apical and basal membranes of differentiated Caco-2 cells in confluent monolayers by immunohistochemistry (Fig 1). The fluorescent immunolabeling was more pronounced on the apical side, as compared to the staining at the basal surface, where the red-stained cell nuclei were situated. This difference between the staining intensity was also visualized by the image of a horizontal section of a representative confluent Caco-2 cell monolayer.

### Effect of cytokines and α-MSH on Caco-2 barrier integrity: transepithelial electrical resistance

**Fig 2:**
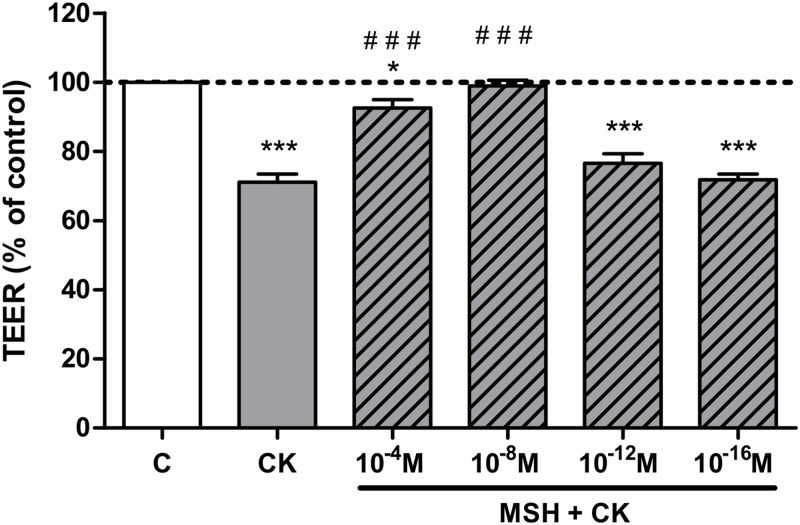
The effect of different concentrations of α-MSH on the electrical resistance of cytokine treated Caco-2 monolayers. Caco-2 monolayers grown on culture inserts were treated with cytokines (CK; 50 ng/ml TNF-α and 25 ng/ml IL-1β) without or with different concentrations of α-MSH (10^−4^ M, 10^−8^ M, 10^−12^ M, and 10^−16^ M) for 24 hours. The control group (C) received culture medium. Transepithelial electrical resistance (TEER) values are shown as percentage of untreated control. Means ± S.D., n = 3; ***P < 0.001, *P < 0.05, ^###^*P*<0.001. *: CK, CK+MSH compared to C; ^#^: CK+MSH compared to CK.

Differentiated Caco-2 monolayers expressed high TEER (700 Ω × cm^2^, n = 24) indicating a tight paracellular barrier. Treatment with a combination of TNF-α and IL-1β cytokines decreased the resistance of Caco-2 monolayers (*P*<0.001; Fig 2). A concentration dependent effect of α-MSH was observed: the 10^−4^ and 10^−8^ M concentrations of α-MSH protected against the cytokine-induced barrier disruption (*P*<0.001), while smaller concentrations had no effect. For further experiments the most effective, 10^−8^ M concentration of α-MSH was selected.

### Effect of cytokines and α-MSH on Caco-2 barrier integrity: permeability for marker molecules

**Fig 3:**
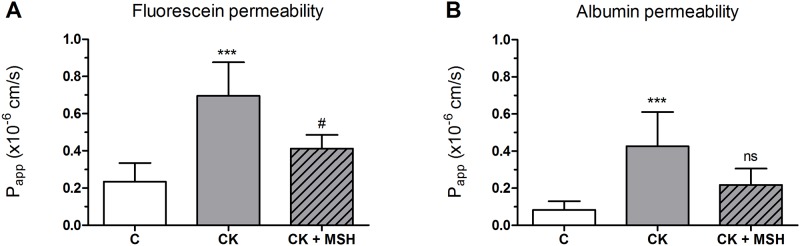
The effect of α-MSH treatment on the permeability of cytokine treated Caco-2 monolayers. Caco-2 monolayers grown on culture inserts were treated with cytokines (CK; 50 ng/ml TNF-α and 25 ng/ml IL-1β) without or with α-MSH (10^−8^ M) for 24 hours, then permeability was measured for fluorescein (A) and albumin (B). The control group (C) received culture medium. Means ± S.D., n = 4–6; ***P < 0.001, ^#^*P* < 0.05. P_app_, apparent permeability coefficient. *: CK, CK+MSH compared to C; ^#^: CK+MSH compared to CK, ns: no significant difference to C or CK.

A low permeability for both small marker fluorescein (1.71 ± 0.17 × 10^−7^ cm/s) and large biomolecule albumin (0.63 ± 0.39 × 10^−7^ cm/s) was measured on the epithelial monolayers. Cytokine treatment significantly enhanced the permeability of the Caco-2 cell layers for both markers (Fig 3). The α-MSH peptide (10^−8^ M) significantly blocked the barrier opening effect of the cytokines for fluorescein.

### Effect of cytokines and α-MSH on Caco-2 barrier integrity: localization of TJ proteins ZO-1 and claudin-4 by immunofluorescence microscopy

**Fig 4:**
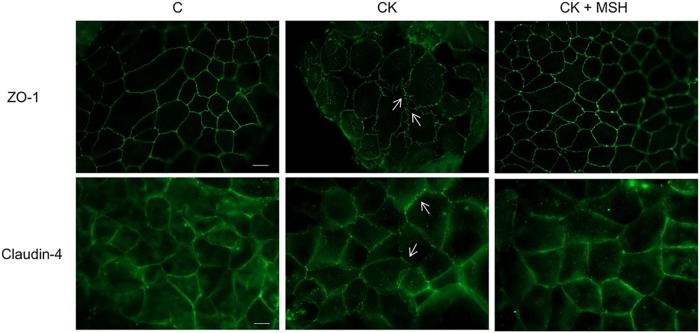
The effect of α-MSH treatment on the immunostaining of tight juntion proteins claudin-4 and ZO-1 in cytokine treated Caco-2 cells. Caco-2 cells were treated with cytokines (CK; 50 ng/ml TNF-α and 25 ng/ml IL-1β) without or with 10^−8^ M α-MSH (CK+MSH) for 24 hours, then immunostained for integral membrane tight junction protein claudin-4 and cytoplasmic linker protein ZO-1. The control group (C) received culture medium. The fluorescent microscopy images demonstrate the expression and organization of the junctional proteins. Scale bar: 10 μm.

The tight paracellular barrier of Caco-2 cells was also demonstrated by the localization of integral membrane TJ protein claudin-4 and cytoplasmic linker protein ZO-1, which appeared at the cell-cell borders in a continuous, belt-like manner (Fig 4). In the control group cell-cell attachment in the monolayers was continuous, without gaps. In cytokine treated cells the pattern of the staining changed: intercellular gaps, fragmented junctional staining and cytoplasmic redistribution of junctional proteins were observed. The immunostaining pattern of TJ proteins in epithelial cells treated with α-MSH was similar to the control group.

### Effect of α-MSH on cytokine-induced NF-κB nuclear translocation in Caco-2 cells

**Fig 5:**
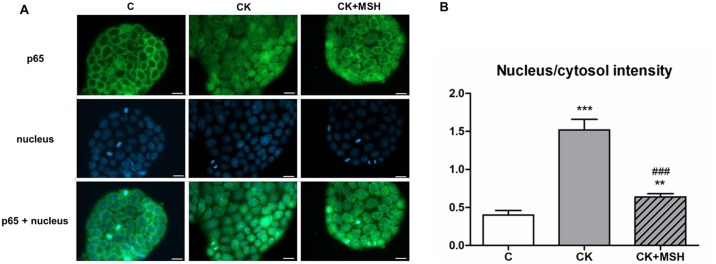
Immunohistochemical staining and analysis of NF-κB activation in cytokine- and α-MSH-treated Caco-2 cells. (**5A**) Caco-2 cells were treated for 30 minutes with culture medium (C), cytokines (CK; 50 ng/ml TNF-α and 25 ng/ml IL-1β), or cytokines and 10^−8^ M α-MSH (CK+MSH). Nuclear localization of the NF-κB p65 subunit was monitored by immunostaining. Cell nuclei were labeled with Hoechst 33342. Green: p65 staining; blue: cell nuclei. Scale bar: 10 μm. (**5B**) Ratio of the fluorescence intensity of the NF-κB immunostaining in cell nuclei and cytoplasm. Means ± S.D., n = 8–10, ***P < 0.001, **P<0.01, ###*P*<0.001. *: CK, CK+MSH compared to C; #: CK+MSH compared to CK.

Translocation of the p65 NF-κB subunit into cell nuclei is a reliable measure of the intensity of an inflammatory reaction at the cellular level. To further analyze the potential effects of α-MSH early NF-κB nuclear translocation was investigated in Caco-2 cells following short term (30 minutes) cytokine treatment (Fig 5A). Inflammatory cytokines induced the appearance of green-stained p65 within nuclei of Caco-2 cells. The nucleus/cytosol intensity ratios of green channel fluorescence provide a numerical display of the cytokine effect [25] with low ratios corresponding to control cells. The increased ratio of signal intensity, combined with co-localization of green and blue signals, reflects inflammatory cytokine-mediated nuclear translocation of p65. The NF-κB nuclear translocation indicated by high nucleus/cytoplasm intensity ratio observed after cytokine treatment was significantly inhibited by 10^−8^ M α-MSH (Fig 5B).

### Effect of cytokines and α-MSH on polarized release of IL-6 and IL-8 from Caco-2 cells

**Fig 6:**
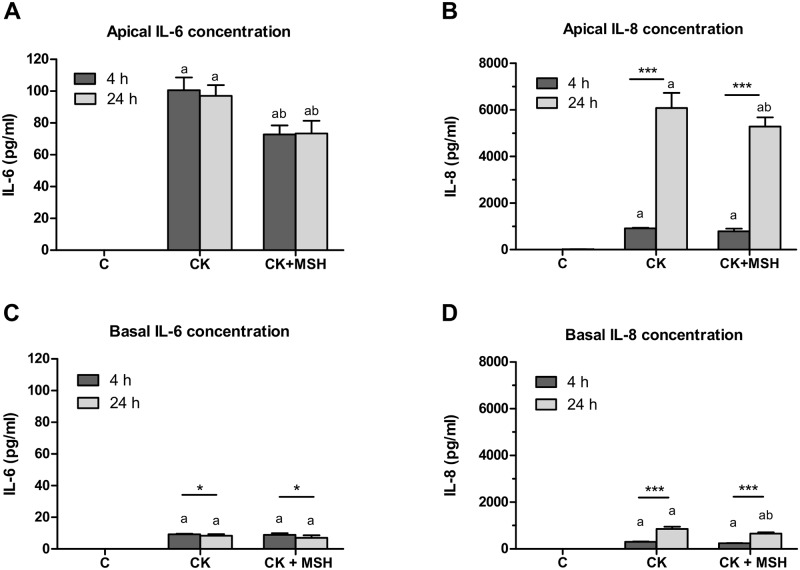
Secretion of IL-6 and IL-8 in Caco-2 cells treated with cytokines and α-MSH. Caco-2 monolayers grown on culture inserts were treated with cytokines (CK; 50 ng/ml TNF-α and 25 ng/ml IL-1β) without or with α-MSH (10^−8^ M) for 4 and 24 hours. The control group (C) received culture medium. Culture medium samples were collected from the apical and basal compartments. The concentration of IL-6 (6A and 6C) and IL-8 (6B and 6D) secreted by Caco-2 cells were measured by multiplexed flow cytometric bead array assays. Means ± S.D., n = 3–5, ***P < 0.001, *P < 0.05; a: CK, CK+ MSH compared to C, *P* < 0.001; b: CK+ MSH compared to CK, *P* < 0.001 [1].

In comparison to unstimulated control cultures, cytokine treatment resulted in significant elevation of IL-6 release into the apical compartments, along with significant α-MSH-dependent suppression of this effect (*P*<0.001) at both 4- and 24-hour time points (Fig 6A). No statistical difference was observed in cytokine expression increase, or in α-MSH-mediated inhibition of this increase between the 4- and 24-hour measurements (Fig 6A). Apical cytokine-induced release of IL-8 increased significantly at both time points relative to the control group, but IL-8 production measured in medium samples collected apically at the 24-hour time point was elevated more than 5-fold (*P*<0.001) relative to that at 4 hours (Fig 6B), but α-MSH had no effect (Fig 6B). Cytokine-induced release of IL-6 and IL-8 into the basal compartments occurred according to a somewhat different pattern from that of the apical medium: although IL-6 release to the basal compartment significantly exceeded that of untreated control cultures (*P*<0.05) (Fig 6C), with no significant difference noted between the 4- and 24-hour time points, α-MSH treatment failed to suppress the basolateral release of IL-6 (Fig 6C). Similarly to apical IL-8 expression responses, cytokine-induced production of IL-8 by Caco-2 monolayers measured from the basal compartments was significantly increased relative to control cells at both the 4-hour and 24-hour time points, but significantly greater at 24 hours (*P*<0.001) (Fig 6D). α-MSH treatment significantly reduced IL-8 expression at 24-hour time points (*P*<0.001) (Fig 6D).

## Discussion

The goal of the present study was to evaluate the protective effect of α-MSH on proinflammatory cytokine-induced Caco-2 monolayer activation and barrier injury.

We demonstrated the polarized expression of MC1R on human Caco-2 epithelial cells with a predominant apical localization (Fig 1) for the first time. Our results are in agreement with the data of Maaser *et al*. who described the constitutive expression of MC1R protein in mouse intestinal epithelial cells [21]. The presence of this receptor provides the basis for the hypothesized direct action of α-MSH on epithelial cells.

A combination of the two most important pro-inflammatory cytokines in gut diseases opened the paracellular barrier in Caco-2 monolayers for both ions measured by TEER (Fig 2) and marker molecules fluorescein and albumin (Fig 3) indicating that there is a barrier leakage for large biomolecules, too. The concentrations of TNF-α and IL-1β were proven to induce barrier damage in Caco-2 cells in previous studies [10,8]. We observed a protective effect of α-MSH against cytokine-induced resistance decrease with a maximal effect at the concentration of 10^−8^ M. This concentration of α-MSH is similar to the concentrations which proved to be effective on other cell types [14,26] and in animal experiments [19,27,28]. The 10^−8^ M concentration of α-MSH we found the most effective corresponds to the physiological plasma concentration of the hormone in humans [29] and in newborn pigs [30]. We also found, that α-MSH-mediated barrier protection is not directly proportional to concentration of the hormone. It was demonstrated earlier that α-MSH, like other peptide hormones, can show bell-shape dose-response curve [31] similarly to our findings. The α-MSH concentration that prevented the drop in TEER was also protective against paracellular barrier opening for fluorescein (Fig 3). Data showing that an α-MSH related tripeptide blocked the reduced TEER induced by cytokines on T84 monolayers, another human intestinal cell line, supports our findings [32].

Since TJ proteins seal the intercellular gaps in epithelial barriers, the disruption of Caco-2 monolayers was also reflected at the level of cell-cell interactions. Two membrane-associated TJ proteins, ZO-1 and claudin-4, were used as representative TJ components. In concordance with the functional measurements of barrier opening, cytokine treatment caused a decreased immunostaining at cellular borders, where punctate and discontinuous staining was observed (Fig 4). In our previous work we also demonstrated morphological changes at cellular junctions in parallel with opening of the paracellular cleft in Caco-2 cells by TJ modulator peptides [12]. In agreement with our observations claudin-4 was described to promote barrier function in different cell cultures, and proinflammatory cytokines decreased its junctional presence and increased the proportion of mobile claudin-4 in the TJ of Caco-2 cells [33,34]. Importantly, claudin-4 expression is downregulated in intestinal biopsies of patients with ulcerative colitis [35], indicating the potential clinical significance of the experimental observations.

The mechanisms that mediate the effect of the proinflammatory cytokines TNF-α and IL-1β on intestinal barrier functions are well studied [7,9,36]. Earlier investigations verified that TNF-α-induced increase in Caco-2 TJ permeability is associated with downregulation of ZO-1 protein expression and alteration in ZO-1 junctional localization. This intracellular process is mediated by NF-κB activation [36]. IL-1β also causes NF-κB activation in Caco-2 cells leading to increased MLCK mRNA and protein expression resulting in elevated junctional permeability [9]. The role of the NF-κB-MLCK pathway was also demonstrated in TNF-α induced TJ opening in Caco-2 monolayers [37]. In the presence of α-MSH we found that the damaging effect of cytokines on TJ morphology was attenuated. Our data are supported by another study where α-MSH prevented the cytokine-induced redistribution of TJ proteins and the opening of the barrier by NF-κB inhibition [14]

Translocation of the NF-κB p65 subunit into cell nuclei is a key event in inflammatory reactions and epithelial barrier opening at the level of signaling pathways [8,38,39]. We could confirm in our study that cytokine treatments induced NF-κB nuclear translocation in Caco-2 epithelial cells, which was inhibited by α-MSH (Fig 5). These results further strengthen the role of αMSH as a potent antiinflammatory molecule [5,14] which exerts its effect by inhibition of the NF-κB transcription factor. Indeed, NF-κB inhibition was found to be central in the protective effect of α-MSH against oxidative stress in IEC-6 rat intestinal cells [40] and against ischemia/reperfusion induced intestinal injury in rats [19,20].

As a downstream effect of NF-κB activation epithelial cells can secrete cytokines in inflammatory conditions further exacerbating gut barrier injury [20]. We found elevated secretion of IL-6 and IL-8 inflammatory cytokines in Caco-2 cells treated with TNF-α and IL-1β (Fig 6). The increased expression of these cytokines was also described in both cultured intestinal cells after inflammatory stimulation [5,6,8] and in a rat model of intestinal injury [20]. We observed that the cytokine-induced IL-6 and IL-8 production was vectorial, with significantly greater apical secretion. The differences between apical and basal IL-6 and IL-8 release from Caco-2 cells are consistent with the results of other investigators [11,5]. Treatment with α-MSH inhibited the apical secretion of IL-6 and the basal release of IL-8. Other studies also support this inhibitory action of α-MSH. KPV, an α-MSH related tripeptide, reduced IL8 expression in Caco-2-BBE cells stimulated by IL-1β [5] or LPS [6]. Notably, KPV and KdPT tripeptides derived from α-MSH were protective not only on injured Caco-2 cultures, but also in mouse and rat models of colitis [32,6]. Importantly, α-MSH also blocked IL-6 protein expression in rat intestinal injury concomitantly with its protective effect [20] underlying the potential therapeutical significance of our findings.

In conclusion, the direct protective effect of α-MSH on pro-inflammatory cytokine induced barrier dysfunction and inflammatory activation in Caco-2 monolayers was investigated for the first time in this study. We demonstrated the presence of major α-MSH receptor MC1 on Caco-2 epithelial cells, and a protective effect of the antiinflammatory hormone on TNF-α and IL-1β treatment induced paracellular barrier opening in parallel with the inhibition of NF-κB nuclear translocation and polarized secretion of other inflammatory cytokines. These findings support the therapeutical potential of α-MSH to restore epithelial barrier integrity in inflammatory gut diseases.
